# Effects of 4(1*H*)-quinolinone derivative, a novel non-nucleotide allosteric purinergic P2Y_**2**_ agonist, on cardiomyocytes in neonatal rats

**DOI:** 10.1038/s41598-017-06481-9

**Published:** 2017-07-20

**Authors:** Kensuke Sakuma, Hideyuki Nakagawa, Tatsuo Oikawa, Masakuni Noda, Shota Ikeda

**Affiliations:** 1 Cardiovascular and Metabolic Drug Discovery Unit, Pharmaceutical Research Division, Takeda Pharmaceutical Company Limited, Fujisawa, Kanagawa Japan; 2 BioMolecular Research Laboratories, Pharmaceutical Research Division, Takeda Pharmaceutical Company Limited, Fujisawa, Kanagawa Japan; 3 Present Address: Regenerative Medicine Unit, Takeda Pharmaceutical Company Limited, 26-1 Muraoka-higashi 2-Chome, Fujisawa, Kanagawa 251-8555 Japan

## Abstract

Purinergic P2Y_**2**_ receptors, G-protein coupled receptors that primarily couple with Gα_q/11_-proteins, are activated equipotently by adenosine-5′-triphosphate (ATP) and uridine-5′-triphosphate. Evidence suggests that P2Y_**2**_ agonists make potential drug candidates for the treatment of cardiovascular diseases. However, selective non-nucleotide, small-molecule P2Y_**2**_ agonists have yet to be developed. In this report, we discuss Compound 89, a novel non-nucleotide allosteric P2Y_**2**_ agonist that was active in signal transduction and gene induction, and in our *in vitro* cardiac hypertrophy model. Compound 89 exhibited selective P2Y_**2**_ agonistic activity and potentiated responses to the endogenous agonist ATP, while exhibiting no agonistic activities for four other Gα_q/11_-coupled human P2Y (hP2Y) receptors and one representative Gα_i/o_-coupled hP2Y_12_ receptor. Its P2Y_**2**_ agonistic effect on mouse P2Y_**2**_ receptors suggested non-species-specific activity. Compound 89 acted as a pure positive allosteric modulator in a Ca^2+^ mobilization assay of neonatal rat cardiomyocytes; it potentiated ATP-induced expression of genes in the nuclear receptor 4A family (negative regulators of hypertrophic stimuli in cardiomyocytes). Additionally, Compound 89 attenuated isoproterenol-induced cardiac hypertrophy, presumably through dose-dependent interaction with pericellular ATP. These results indicate that Compound 89 is potentially efficacious against cardiomyocytes and therefore a good proof-of-concept tool for elucidating the therapeutic potential of P2Y_2_ activation in various cardiovascular diseases.

## Introduction

P2Y receptors, G-protein coupled receptors (GPCRs) that are activated by extracellular adenine/uridine nucleotides and nucleotide sugars, mediate various physiological functions in virtually all tissues and cells^1^. Studies have shown that among them, P2Y_2_ receptors are broadly and highly expressed in the eyes, lungs, intestine, immune system, and skeletal muscles, as well as tissues associated with cardiovascular homeostasis (i.e. blood vessels, heart, kidneys, and brain)^2–5^. Increasing evidence indicates that P2Y_**2**_ receptors could function as cell-surface ‘mechano-sensors’ for the autocrine or paracrine release of adenosine-5′-triphosphate (ATP) and/or uridine-5′-triphosphate (UTP) from different cellular sources^6–9^ in order to primarily mediate Gα_q/11_ signalling, resulting in downstream transcriptional regulation via nuclear inositol 1,4,5-trisphosphate receptor-induced local Ca^2+^ signalling in muscle fibres and cardiac myocytes^10–12^. Such regulation of transcriptional activity through local Ca^2+^ signal transduction is known as excitation-transcription coupling, as opposed to the excitation-contraction coupling that links global increases in intracellular Ca^2+^ more closely with muscle contractions.

A growing number of studies have suggested that P2Y_**2**_ receptors potentially improve cardiovascular dysfunction in various ways, such as through endogenous UTP-mediated cardioprotection during ischemia^13, 14^, ATP-mediated antihypertrophic effects in the heart^2, 15^, control of shear stress-induced blood pressure and vessel arteriogenesis^16, 17^, and maintenance of normal renal functions^18^. Thus, the pharmacological activation of P2Y_**2**_ receptors can provide symptomatic relief of cardiovascular dysfunction.

Several nucleotide-based P2Y_**2**_ agonists and an allosteric partial agonist have been identified and synthesized through extensive library screening in conjunction with molecular modelling^19–21^. One of the most advanced compounds is diquafosol, an analogue of UTP for the treatment of dry eye^22^. However, that compound is not suitable for oral administration because of high polarity and low intestinal absorption. Non-nucleotide P2Y_**2**_ antagonists such as Reactive Blue 2^23^, suramin^24^, AR-C118925^25, 26^ (a thiouracil derivative), anilinoanthraquinone derivatives^27^, and some flavonoids^28^ have been reported, but novel non-nucleotide P2Y_2_ selective agonists—compounds that could help elucidate the effects of P2Y_2_ activation in peripheral tissues—had yet to be discovered until now. Indeed, while the recent rapid advances in GPCR and ion channel crystallography have enhanced the practical understanding of purinergic receptors such as A_2A_AR, P2X_4_, P2Y_1_, and P2Y_12_^29–34^, a structure-based approach to ligand design that is capable of predicting ligand selectivity and functionality remains under development^35^.

In this study, we investigated a novel highly selective non-nucleotide P2Y_2_ allosteric agonist: Compound 89, a 4(1*H*)-quinolinone derivative with the chemical name 2-((ethyl(4-fluorobenzyl)amino)methyl)-7,8-dimethylquinolin-4(1*H*)-one. Compound 89 was selected from our compound library based on its effect on Ca^2+^ mobilization in the 1321N1 human astrocytoma cell line. The 1321N1cells are useful for identifying P2Y isoform-specific agents, because they lack P2 receptors and are unresponsive to nucleotides^36, 37^. We also studied the effects of Compound 89 on Ca^2+^ signalling, nuclear receptor 4A (NR4A) gene induction, and isoproterenol-induced cardiac hypertrophy in neonatal rat cardiomyocytes (NRCMs).

## Results

### Compound 89 is a highly selective agonist for P2Y_2_ receptors relative to other P2Y receptors in P2Y-overexpressing 1321N1 cells

**Figure 1:**
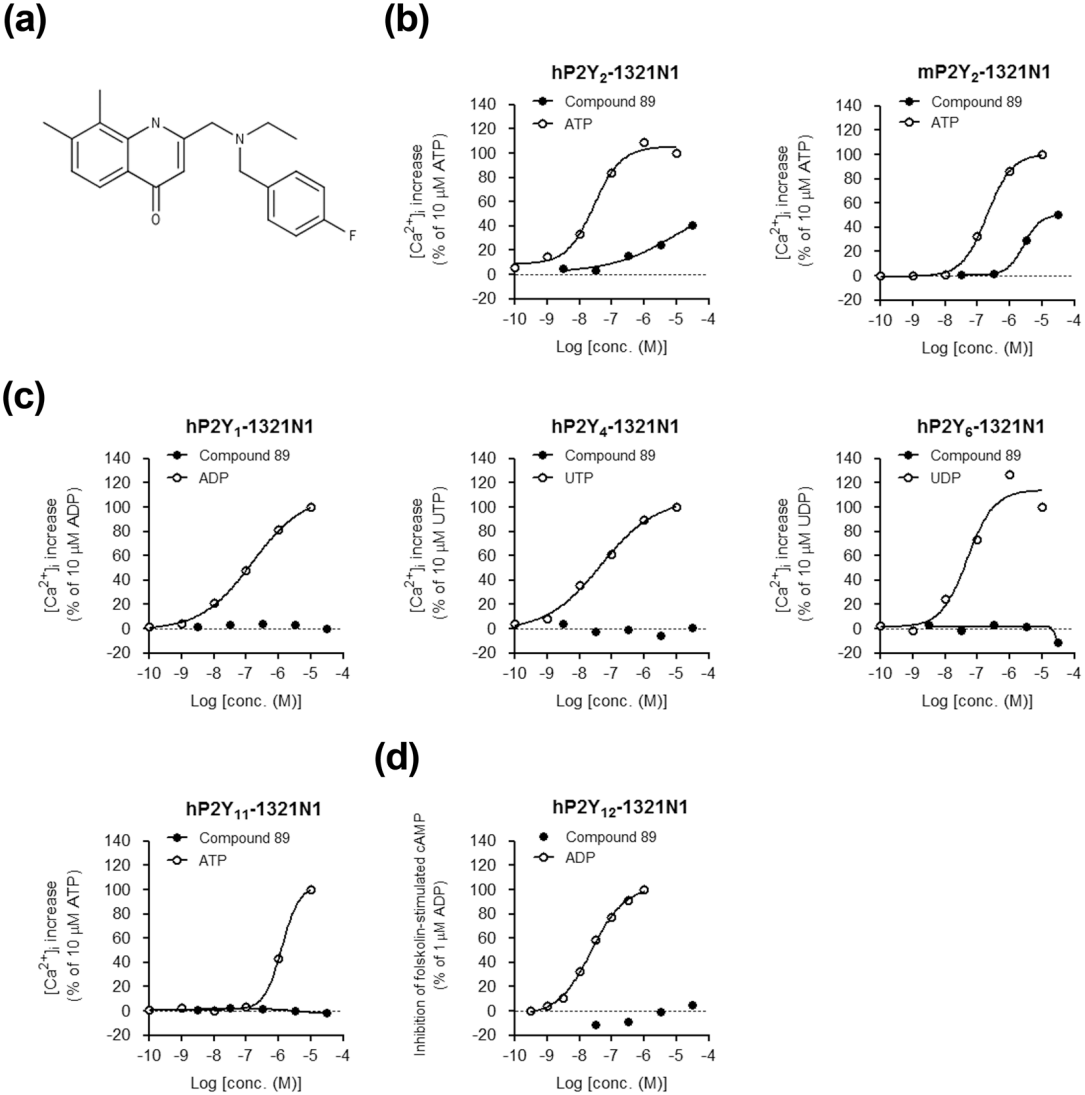
Compound 89 displays selective agonist activity for P2Y_2_ in P2Y-overexpressing 1321N1 cells. (**a**) The chemical structure of the 4(1*H*)-quinolinone derivative Compound 89: 2-((ethyl(4-fluorobenzyl)amino)methyl)-7,8-dimethylquinolin-4(1*H*)-one. (**b**) Agonistic activity against human and mouse P2Y_2_ receptors in a FLIPR Ca^2+^ mobilization assay using 1321N1 cells that were transiently-expressing hP2Y_2_ receptors (hP2Y_2_-1321N1) and stably-expressing mP2Y_2_ receptors (mP2Y_2_-1321N1). The activity of 10 μM ATP was normalized to 100%. (**c**) Agonistic activity against human P2Y_1_, P2Y_4_, P2Y_6_, and P2Y_11_ receptors in FLIPR Ca^2+^ mobilization assays utilising their respective transiently-expressing 1321N1 cells. The agonistic activity of 10 μM ADP for hP2Y_1_, UTP for hP2Y_4_, UDP for hP2Y_6_, and ATP for hP2Y_11_ was normalized to 100%. (**d**) Agonistic activity against human P2Y_12_ receptor in a cAMP accumulation assay using stably-expressing hP2Y_12_-1321N1 cells. The activity of 1 μM ADP was normalized to 100%. All data points represent the mean in duplicate or quadruplicate and comparable results were obtained from another independent experiment.

By using the 1321N1 human astrocytoma cell line, we were able to identify Compound 89 (Fig. 1a) from our compound library as a potential P2Y_2_ selective agonist. In our study, a FLIPR Ca^2+^ mobilization assay of hP2Y_2_–1321N1 cells revealed that Compound 89 had agonistic activity against human P2Y_2_, although it was less potent than ATP (Fig. 1b). Compound 89 also exhibited agonistic activity against mouse P2Y_2_ in mP2Y_2_-1321N1 cells (Fig. 1b), suggesting that agonistic activity is conserved across species. The calculated EC_50_ values (potency) for Compound 89 were 10.5 μM and 2.7 μM for human and mouse P2Y_2_ receptors, respectively; and the extrapolated maximum efficacies for Compound 89 with respect to 10 μM ATP were 65.8% and 50.9% for human and mouse P2Y_2_ receptors, respectively (Fig. 1b).

The selectivity of Compound 89 for P2Y_2_ was analysed in hP2Ys-1321N1 cells. The P2Y receptors were grouped into two subgroups: five Gα_q/11_-coupled receptors (P2Y_1_, P2Y_2_, P2Y_4_, P2Y_6_, and P2Y_11_) and three Gα_i/o_-coupled receptors (P2Y_12_, P2Y_13_, and P2Y_14_)^1, 11^. While endogenous nucleotide ligands showed clear dose-response patterns, Compound 89 (at up to 30 µM) lacked agonistic activity against the remaining four Gα_q/11_-coupled hP2Y receptors in the FLIPR Ca^2+^ mobilization assay (P2Y_1_, P2Y_4_, P2Y_6_ and P2Y_11_), and one representative Gα_i/o_-coupled hP2Y_12_ receptor in the cAMP accumulation assay (Fig. 1c and d). Thus, Compound 89 appeared to selectively activate hP2Y_2_ receptors. For comparison, transfection experiments confirmed that 10 μM concentrations of endogenous P2Y ligands (ATP, UTP, adenosine-5′-diphosphate [ADP], and uridine-5′-diphosphate [UDP]) lacked activity in empty vector-transfected parental 1321N1 cells (data not shown), which is consistent with the previous reports^36, 37^.

### Compound 89 exhibits allosteric modulation of ATP-induced Ca^2+^ mobilization in P2Y_2_-overexpressing 1321N1 cells and neonatal rat cardiomyocytes

**Figure 2:**
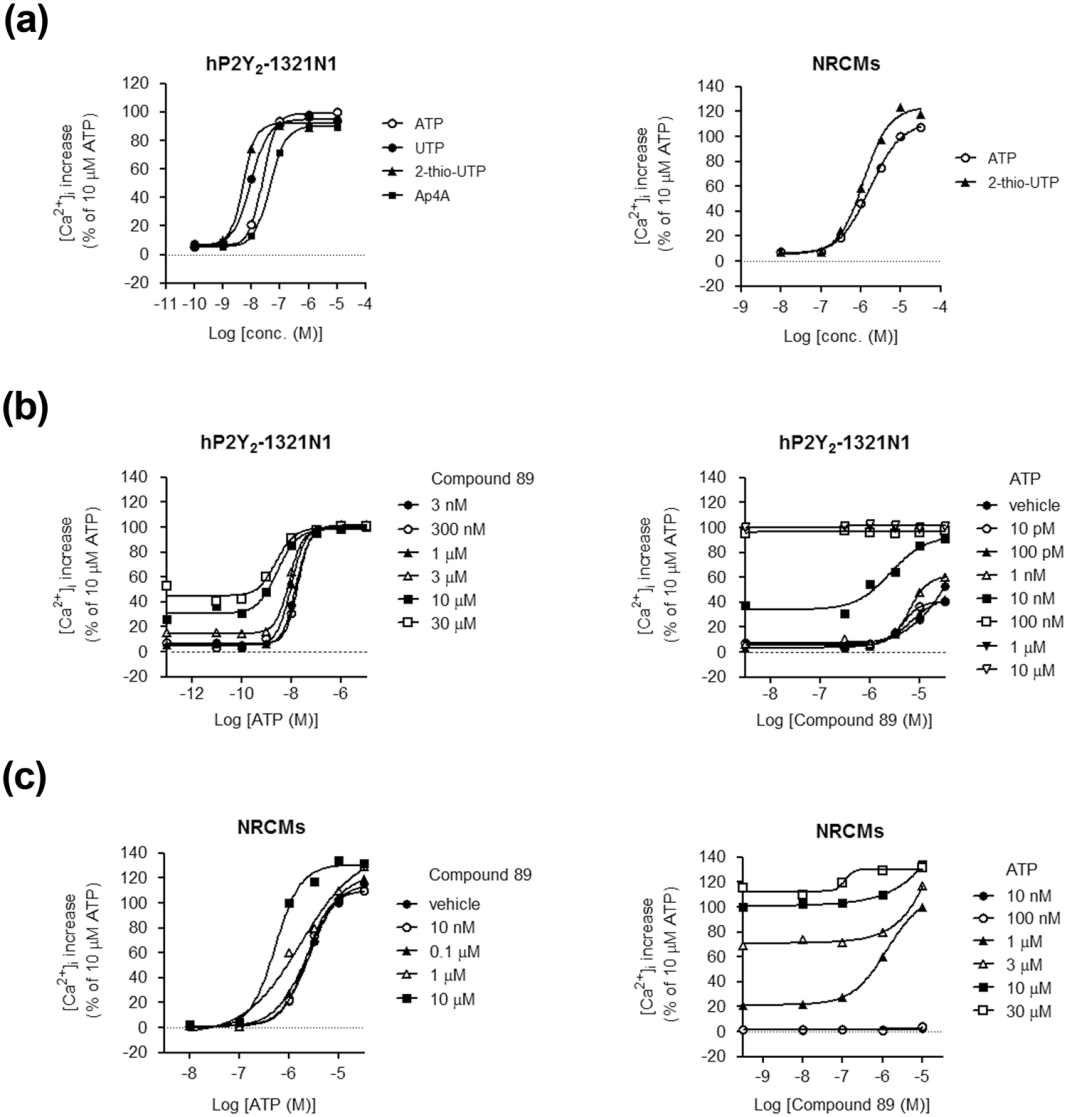
Compound 89 exhibits allosteric modulatory effects on ATP-induced Ca^2+^ mobilization in P2Y_2_-overexpressing 1321N1 cells and in cardiomyocytes. (**a**) Concentration-response curves of physiological agonists in a FLIPR Ca^2+^ mobilization assay using hP2Y_2_ stably-expressing 1321N1 cells and NRCMs. (**b**) Allosteric modulatory effect of Compound 89 on the ATP concentration-response curve (*left*), and allosteric modulatory effect of ATP on the Compound 89 concentration-response curve (*right*) in hP2Y_2_ stably-expressing 1321N1 cells. (**c**) Allosteric modulatory effect of Compound 89 on the ATP concentration-response curve (*left*), and the allosteric modulatory effect of ATP on the Compound 89 concentration-response curve (*right*) in NRCMs. The activity of 10 μM ATP was normalized to 100%. All data points represent the mean of experiments performed in duplicate or triplicate, and comparable results were obtained by another independent experiment.

To explore the interaction between Compound 89 and endogenous ATP during P2Y_2_ activation, we investigated how Compound 89 affects the agonistic activity of ATP and vice versa. In a FLIPR Ca^2+^ mobilization assay utilizing hP2Y_2_-1321N1 cells, the concentration-response curves of three physiological agonists (ATP, UTP, and diadenosine tetraphosphate [Ap4A]) and the synthesized agonist 2-thio-UTP (a P2Y_2_ preferential agonist)^38, 39^ were evaluated (Fig. 2a, *left*). Addition of Compound 89 caused an upward and leftward shift in the ATP concentration-response curve, implying positive allosteric modulation of P2Y_2_ by ATP (an orthosteric agonist) and Compound 89 (Fig. 2b, *left*). The EC_50_ values for ATP decreased from 15.1 nM to 2.4 nM in the presence of 3 nM to 30 µM Compound 89, respectively. In comparison, addition of increasing concentrations of ATP caused a progressive upward and leftward shift followed by an upper plateau in the Compound 89 concentration-response curve. The EC_50_ value for Compound 89 decreased from 53.9 μM in the absence of ATP, to 2.5 μM in the presence of 10 nM ATP (Fig. 2b, *right*). Similar findings were observed with the endogenous agonists UTP (an orthosteric agonist) and Ap4A (Supplementary Fig. S1). These results demonstrate that in hP2Y_2_-expressing human astrocytoma 1321N1 cells, Compound 89 behaves as a probe-independent, highly selective P2Y_2_ positive allosteric modulator with intrinsic agonistic activity.

We next examined the potency of Compound 89 under physiological conditions using NRCMs. In a FLIPR Ca^2+^ mobilization assay, ATP and 2-thio-UTP exhibited comparable concentration-response curves and maximum efficacies (Fig. 2a, *right*), implying that NRCMs can be used to estimate Gα_q/11_-mediated Ca^2+^ signalling downstream from P2Y_2_. Then, we evaluated how the agonistic activity of ATP was affected by the presence of Compound 89 in NRCMs. Compound 89 caused a left- and upward shift in the ATP concentration-response curve, indicating that the compound behaves as a positive allosteric modulator of P2Y_2_ even in NRCMs (Fig. 2c, *left*). In addition, the EC_50_ values for ATP decreased from 2.6 μM in the absence of Compound 89, to 0.53 μM in the presence of 10 μM Compound 89; and the maximum efficacy in the presence of 10 μM Compound 89 reached 130.7%. Likewise, a progressive upward and leftward shift in the Compound 89 concentration-response curve was obtained by the addition of increasing concentrations of ATP (Fig. 2c, *right*). The EC_50_ values for Compound 89 decreased from 1.3 μM in the presence of 1 μM ATP, to 0.11 μM in the presence of 30 μM ATP; and the maximum efficacy in the presence of 30 μM ATP reached 130.5%. Of note, Compound 89 displayed no intrinsic agonistic activity in NRCMs, which was not the case in hP2Y_2_-1321N1 cells (Fig. 2b and c, *right*), suggesting that the compound acts as a pure P2Y_2_ positive allosteric modulator in NRCMs.

### Compound 89 allosterically enhances ATP-induced gene expression in cardiomyocytes

**Figure 3:**
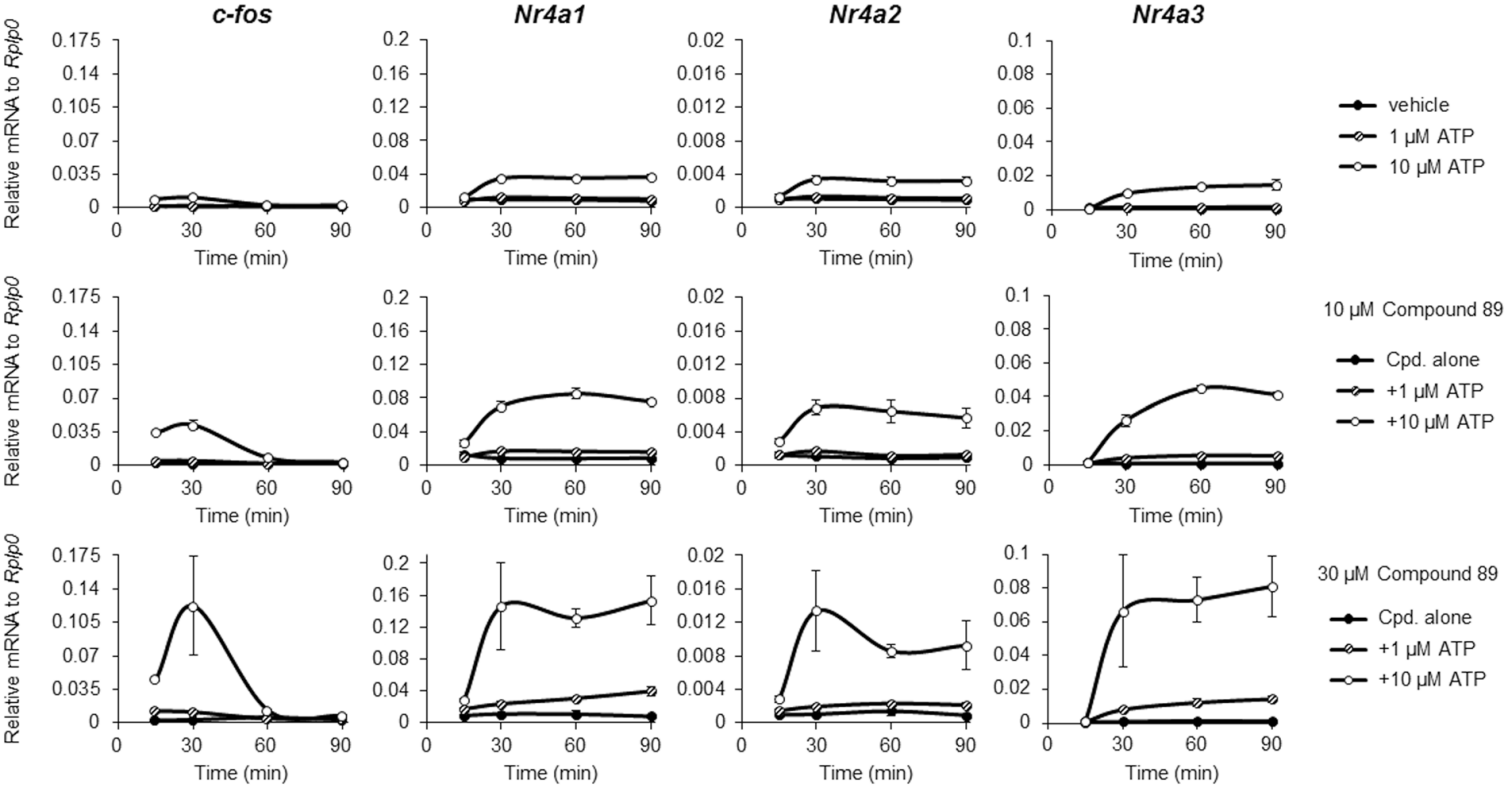
Compound 89 allosterically potentiates ATP-induced changes in IEG gene expression in cardiomyocytes. Gene expression changes of IEGs, *c-fos* and the NR4A subfamily, in a time-dependent manner. Inductions by ATP (0, 1 or 10 μM) in the presence of Compound 89 (0, 10 or 30 μM) were plotted. Expression levels are normalized to *Rplp0* and shown as means ± SEM (n = 3).

We sought to determine how P2Y_2_ activation in cardiomyocytes regulates the expression of the NR4A subfamily of orphan nuclear receptors, consisting of NR4A1 (Nur77), NR4A2 (Nurr1), and NR4A3 (Nor-1). These receptors, previously reported as negative-feedback regulators for hypertrophic stimuli in NRCMs^40^, are rapidly induced by a broad spectrum of stimuli^41^. We stimulated the receptors with endogenous agonist ATP alone and in combination with Compound 89 over 90 min and examined the time-course changes in the mRNA expression of *Nr4a1*, *Nr4a2*, *Nr4a3* and another immediate early gene (IEG), *c-fos*. Stimulation with ATP at 10 μM increased the expression of all *Nr4a* genes steadily after 30 min, while *c-fos* expression was transiently induced with a single peak evident at 30 min (Fig. 3, *upper*). Co-administration of the compound at 10 and 30 μM enhanced ATP-induced gene expression changes in a concentration-dependent manner (Fig. 3, *middle* and *lower*). Interestingly, Compound 89 itself did not induce any changes in gene expression. These results suggest that Compound 89 serves as a pure positive allosteric regulator of P2Y_2_ that potentiates gene expression changes induced by ATP, which is consistent with results from our signalling assay (Fig. 2c).

### Compound 89 reverses cardiac hypertrophy induced by β-adrenergic agonists in cardiac myocytes

**Figure 4:**
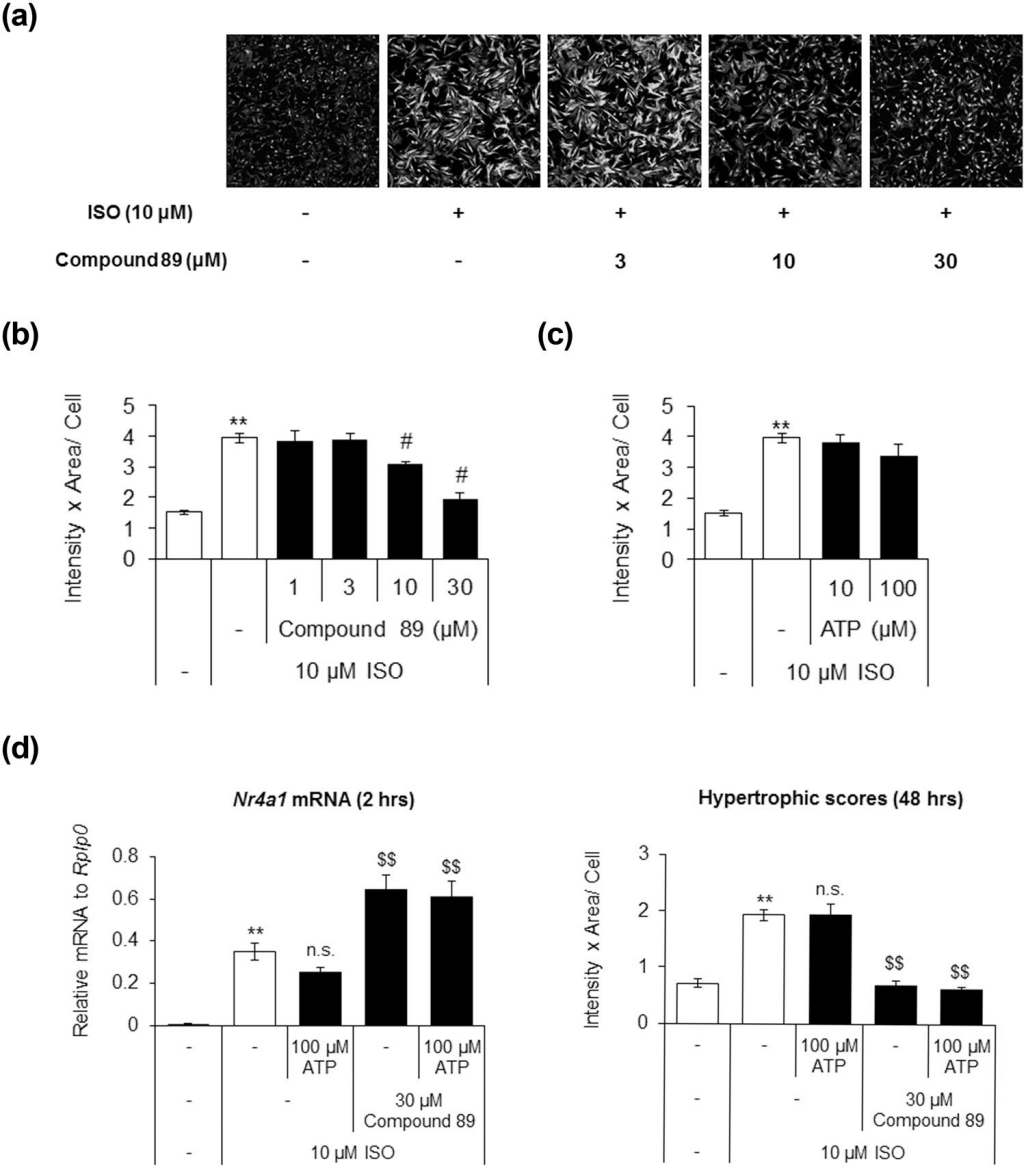
Compound 89 exerts anti-hypertrophic effect in cardiomyocytes. (**a**) Fluorescent microscopic images of filamentous actin in NRCMs labelled with phalloidin-FITC, and imaged with 10 × 0.45 NA objective on a 96-well plate. (**b**,**c**) Quantification of the images and effects of (**b**) Compound 89 (1 to 30 μM) and (**c**) ATP (10 and 100 μM). Hypertrophic scores were calculated as the product of FITC-fluorescence intensity and cell area per cell. Data are shown as means ± SD (n = 3). ^#^ *P* < 0.025 versus 10 μM isoproterenol (ISO) alone, one-tailed Williams’ test. **P < 0.01 versus vehicle, Student’s t-test. (**d**) Changes in expression of *Nr4a1* mRNA at 2 h, and hypertrophic scores at 48 h after P2Y_2_ activation by 100 μM ATP and/or 30 μM Compound 89 in the presence of 10 μM isoproterenol. Data are shown as mean ± SD (n = 4). **P < 0.01 versus vehicle, Aspin-Welch test (*Nr4a1* mRNA) or Student’s t-test (hypertrophic scores). ^$$^ *P* < 0.01 versus 10 μM isoproterenol (ISO) alone, Dunnett’s test. n.s., not significant.

Earlier studies have demonstrated that exogenous ATP stimulation and NR4A1 induction have anti-hypertrophic effects on cardiac myocytes^15, 40^. Because Compound 89 can potentiate the expression of genes in the NR4A family, we speculated that it could exert an anti-hypertrophic effect in cardiac hypertrophy. To test this hypothesis, we evaluated the effects of Compound 89 on isoproterenol-induced cardiac hypertrophy in NRCMs. Before starting, we confirmed that Compound 89 does not affect β-adrenergic receptor-mediated cAMP signalling in parental 1321N1 cells, which have been reported to express endogenous β-adrenergic receptors^42, 43^ (Supplementary Fig. S2). Isoproterenol-induced cardiac hypertrophy was attenuated by Compound 89 in a concentration-dependent manner (Fig. 4a and b). In comparison, treatment with ATP produced no significant effects (*P* = 0.568, Fig. 4c). To interpret these results, we also analysed the changes in expression of *Nr4a1* mRNA at 2 h, as well as the resulting hypertrophic scores at 48 h after the start of stimulation. As shown in Fig. 4d, the β-adrenergic agonist isoproterenol significantly induced *Nr4a1* at 2 h (*P* = 5 × 10^−4^), in line with a previous report^40^. Induction of *Nr4a1* was greater with Compound 89 than with isoproterenol alone, while ATP did not affect induction (Fig. 4d). These changes in expression corresponded well with alterations in cardiac hypertrophy. Complete attenuation of hypertrophy was observed after 48 h of treatment with Compound 89 (Fig. 4d), suggesting that early induction of NR4As determines the antihypertrophic phenotype for isoproterenol-induced cardiac hypertrophy.

## Discussion

In this study, we demonstrated that Compound 89 is an allosteric agonist for P2Y_2_ that potentiates ATP-induced downstream responses, including Ca^2+^ influx and gene induction. Three key features distinguish Compound 89 from other compounds: structure, specificity, and allosteric agonistic properties. First, Compound 89 has a 4(1*H*)-quinolinone-based non-nucleotide structure (Fig. 1a). Given the difficulties of producing nucleotide analogues in medicinal chemistry (i.e. difficulties with high yield, reproducible synthesis, purification, etc.)^44^, the development of non-nucleotide P2Ys ligands is worth pursuing. Second, Compound 89 was capable of distinguishing between human P2Y_2_ and P2Y_4_ (Fig. 1b and c), the two closest phylogenetic subtypes^1^. In addition, its agonistic activity against mouse P2Y_2_ receptors (Fig. 1b) implied that Compound 89’s binding capacity is not species-specific, a speculation supported by the high (90%) similarity in amino acid sequence among human, mouse, and rat P2Y_2_ receptors. Finally, although Compound 89 displayed partial agonistic activity as well as positive allosteric modulation of ATP responses in P2Y_2_-overexpressing 1321N1 cells, it served as a purely positive allosteric modulator in NRCMs (Fig. 2b and c). Considering that partial agonistic activity is dependent on receptor expression levels and coupling efficacy^45–47^, the difference may be due to the variation in P2Y_2_ expression levels between hP2Y_2_-1321N1 cells and NRCMs.

A number of allosteric modulators have been discovered for class A GPCRs, such as adenosine receptors, free fatty acid receptors, and muscarinic receptors, some of which are supported by the X-ray co-crystallography^48–50^. In addition, a series of nucleotide-derivative P2Y_2_ allosteric agonists have previously been reported^21^; but as a non-nucleotide allosteric modulator of P2Y_2_, Compound 89 is in a potentially novel class. In a study by Van Poecke *et al*.^21^, a series of synthesized phosphonate analogues at their highest concentrations induced ~10% of the maximum response of UTP in hP2Y_4_ stably expressing 1321N1 cells, suggesting weak agonistic activity against human P2Y_4_ receptors. In our present study, Compound 89 showed agonistic activity against P2Y_2_, but not against P2Y_1_, P2Y_4_, P2Y_6_, and P2Y_11_ at up to 30 μM (Fig. 1b and c). Although subtle differences between assay systems exist, our results indicate that Compound 89 may be a truly selective P2Y_2_ allosteric agonist.

To clarify the role of ATP/P2Y_2_-mediated signalling in NRCMs, we investigated the regulation of putative target genes downstream of P2Y_2_. The NR4A subfamily, consisting of NR4A1 (Nur77), NR4A2 (Nurr1), and NR4A3 (Nor-1), is a group of transcriptional regulators involved in the control of tissue metabolism and energy balance. The functional significance of the NR4A family in the heart has not been elucidated well in comparison to other tissues such as the liver, skeletal muscles, and adipose tissues. However, the NR4A family has been implicated in cardiac remodelling that occurs in response to β-adrenergic stimulation^51^. In addition, NR4A1 has recently been reported to function as a negative-feedback regulator for hypertrophic stimuli in cardiomyocytes^40^. Although NR4As are rapidly and transiently induced by diverse stimuli such as receptor agonists, activators for intracellular mediators, mechanical and chemical factors, and exercise^41^, P2Y_2_-mediated upregulation of NR4As has not been fully described. Given the multiplicity of NR4A stimulators and their high sensitivity, it is surprising that Compound 89 alone did not induce expression of NR4As, but only enhanced ATP-induced NR4A responses, thus reflecting Compound 89’s pharmacological property as a pure positive allosteric modulator of P2Y_2_ in NRCMs (Figs 2c and 3).

Compound 89 inhibited isoproterenol-induced hypertrophy in NRCMs in a concentration-dependent manner (Fig. 4a and b). Mechanistically, Zheng, *et al*.^15^ previously reported that ATP signalling, presumably at least partly mediated via P2Y_2_, could induce transcriptional activation that is somewhat distinct from that induced by other Gα_q/11_-mediating neurohumoral hypertrophic factors such as norepinephrine and phenylephrine. Such a difference would result in a unique anti-hypertrophic effect, although further mechanistic analysis would be required. In the present study, Compound 89’s anti-hypertrophic activity was not affected by the presence of ATP (Fig. 4d). One possible explanation is that ATP released by isoproterenol-induced contractions of NRCMs reached a plateau in the pericellular area, thus abrogating the effect of exogenous ATP stimulation. This hypothesis is supported by the fact that intracellular ATP is released into pericellular space in response to various mechanical and biochemical stimuli^6^. Aside from skeletal and cardiac muscles themselves, multiple potential sources exist for interstitial ATP including the surrounding nerve endings, blood cells, and endothelium, via vesicular and conductive mechanisms of exocytosis, apoptosis, Cl^−^ channels and pannexin hemichannels^52, 53^.

To date, the autocrine release of ATP via pannexin 1 hemichannels from cardiac myocytes has been substantiated through the combined use of cultured cardiac myocytes and bioluminescent probes^54, 55^. Those reports have estimated extracellular ATP levels in the mid-nanomolar range, which is likely to fall short of EC_50_ values (low micromolar range; Fig. 2a). However, the actual ATP concentration very close to the cell membrane surface could be much higher, although almost impossible to measure accurately considering the nucleotide-degrading system based on ectonucleotidases such as CD39 and CD73^8^. ATeams, a new class of genetically encoded fluorescent ATP indicators, might help to overcome these limitations^56^. At any rate, it is possible that greater induction of NR4As by a combination of β-adrenergic stimulation and P2Y_2_ allosteric potentiation resulted in the attenuation of cardiac hypertrophy shown in Fig. 4d.

In summary, our results demonstrate that Compound 89, a 4(1*H*)-quinolinone derivative, is a potentially novel non-nucleotide allosteric P2Y_2_ agonist that inhibits the development of β-adrenergic agonist–induced cardiac hypertrophy through activation of NR4As. Our data provided clues for the development of a new series of P2Y_2_ allosteric agonists, which will help elucidate the therapeutic potential of P2Y_2_ activation in various cardiovascular diseases.

## Materials and Methods

### Reagents

Compound 89, also known as 2-((ethyl(4-fluorobenzyl)amino)methyl)-7,8-dimethylquinolin-4(1*H*)-one, was purchased from ChemDiv (San Diego, USA). Adenosine-5′-diphosphate monosodium salt, ATP disodium salt, UDP monosodium salt, and isoproterenol hydrochloride were purchased from Wako Pure Chemicals (Osaka, Japan). Uridine-5′-triphosphate was purchased from Sigma-Aldrich Japan (Tokyo, Japan). 2-Thio-UTP was purchased from Tocris Bioscience (Bristol, UK). Diadenosine 5′,5′″-P^1^,P^4^- tetraphosphate was purchased from Jena Bioscience (Jena, Germany).

### Animals

Newborn pups (2–4 days old) born from female Wistar rats (CLEA Japan) were used for primary culturing of cardiomyocytes. The animals were housed in an environment with a 12 h dark-light cycle (lights on at 7:00 a.m.), and fed food (CE-2, CLEA Japan) and tap water *ad libitum*. All experiments were performed in accordance with protocols reviewed and approved by the Institutional Animal Care and Use Committee (IACUC) at the Takeda Pharmaceutical Company, Ltd.

### Cell preparation

The human astrocytoma 1321N1 cell lines, which stably express human P2Y_2_, P2Y_12_, and mouse P2Y_2_ receptors (i.e. hP2Y_2_-, hP2Y_12_-, and mP2Y_2_-1321N1 cells), were established at the Takeda Pharmaceutical Company (Fujisawa, Japan). The 1321N1 cells were grown in Dulbecco’s modified Eagle’s medium (DMEM) containing 10% heat-inactivated foetal bovine serum, 100 IU/mL penicillin, and 100 μg/mL streptomycin (Thermo Fisher Scientific Japan). The aminoglycoside G-418 (0.5 mg/mL; Thermo Fisher Scientific) was added to culture media only when selection was made. For transient expression studies, human P2Ys/pcDNA3.1(+) plasmids were transfected into 1321N1 cells using Lipofectamine 3000 (Thermo Fisher Scientific).

Neonatal rat cardiomyocytes were isolated from the hearts of Wistar rats (2–4 days old) through enzymatic dissociation. In brief, isolated hearts were minced and digested by stirring in 1% collagenase II (Worthington, NJ, USA) at 37 °C for 30 min, then cultured at 37 °C in a humidified atmosphere containing 5% CO_2_ and 95% air for 90 min to allow fibroblasts to attach to the dish. Floating NRCMs were then collected and seeded in a collagen I-coated 96-well plate using DMEM/Nutrient Mixture F-12 supplemented with 10% foetal bovine serum, penicillin (100 IU/mL), and streptomycin (100 μg/mL) until use.

### Ca^2+^ mobilization assay

To assess intracellular Ca^2+^ levels in P2Ys-expressing 1321N1 cell lines, cells were seeded in 96- or 384-well plates, and incubated at 37 °C for 1 h in HEPES-buffered Hank’s balanced salt solution (HBSS; pH = 7.4) containing 2.5 mM probenecid, 0.1% fatty acid-free bovine serum albumin, and Ca^2+^ indicator dyes: a wash-type acetoxymethyl ester of Fluo-4 (Fluo-4 AM; Dojindo, Japan) or a homogenous no-wash type dye (Fluorometric Imaging Plate Reader [FLIPR] calcium assay kit; Molecular Devices Japan). For NRCMs, isolated cells were seeded in collagen I-coated 96-well plates and a FLIPR calcium assay kit was used to reduce cell damage. Intracellular Ca^2+^ levels over time were measured before and after the addition of ligands by using a FLIPR. Fluorescence intensity was defined as the difference between the maximum and minimum values during the measurement period, normalized to the intensity produced by submaximal concentrations of endogenous nucleotide ligands for individual P2Y receptors as indicated in each figure. The EC_50_ and E_max_ values for each curve were determined through data analysis and curve-fitting using a 4-parameter logistic equation in Prism 5 software (GraphPad Software, CA, USA).

### cAMP accumulation assay

To measure the intracellular cyclic adenosine monophosphate (cAMP) levels in hP2Y_12_-1321N1 cells, a cAMP cell-based Dynamic 2 (d2) assay kit based on homogeneous time resolved fluorescence (HTRF Technology; Cisbio Bioassays, France) was used according to the attached manual. Suspended cells were stimulated with 1 μM forskolin and ligands, followed by incubation for 30 min at room temperature. Then, cAMP-d2 and anti-cAMP-cryptate reagents in lysis buffer were added. The lysate was incubated for 60 min at room temperature and then analysed using an EnVision plate reader (Thermo Fisher Scientific).

### mRNA expression analysis

Neonatal rat cardiomyocytes were seeded in a collagen I-coated 96-well plate, then cultured and maintained for a week before the ligand stimulation assay. The culture medium was replaced by HBSS buffer containing 20 mM HEPES and then stimulated with the indicated ligands for 15 to 90 min. After stimulation, cDNA samples were synthesized from lysates using TaqMan Gene Expression Cells-to-Ct kits (Thermo Fisher Scientific) according to the manufacturer’s instructions, followed by quantitative real-time polymerase chain reaction analysis using a Prism 7900HT sequence detector (Thermo Fisher Scientific). Thermal cycling parameters were 2 min at 50 °C and 10 min at 95 °C, followed by 40 cycles at 95 °C for 15 sec and 60 °C for 1 min. The mRNA levels were analysed with the comparative Ct method (2^−ΔΔCt^) using *Rplp0* as the housekeeping gene. The TaqMan Gene Expression assays (Thermo Fisher Scientific) used herein were as follows: Rn01533237_m1 (*Nr4a1*), Rn00570936_m1 (*Nr4a2*), Rn01354012_m1 (*Nr4a3*), Rn02396759_m1 (*c-fos*), and Rn03302271_gH (*Rplp0*).

### Cardiac hypertrophy measurements

Neonatal rat cardiomyocytes were seeded in a 96-well collagen I plate at a density of 5000 cells/well. They were incubated overnight and then transferred to a serum-free medium for one day. Next, the cells were treated with the indicated concentrations of compounds and 10 μM isoproterenol, and then incubated for 2 days. To quantify cardiomyocyte hypertrophy via filamentous actin staining, NRCMs were fixed with 4% paraformaldehyde (Wako) and permeabilized with 0.1% Triton X-100 (MP Biomedicals, France), followed by blocking with 1% bovine serum albumin (Wako) and staining with 2 μM phalloidin–fluorescein isothiocyanate (phalloidin-FITC; Sigma-Aldrich, Japan) and 10 μM Hoechst 33258 (Dojindo). Quantification of FITC-fluorescence intensity and cell number was performed using an IN Cell Analyzer 6000 (GE Healthcare Japan). The signals from obtained images were quantitated by means of a customized image analysis algorithm using the IN Cell Developer Toolbox (GE Healthcare Japan). Hypertrophic scores were calculated as the product of FITC-fluorescence intensity and cell area per cell as programmed by GE Healthcare technical support experts.

### Data analysis and statistics

The dose-response relationships in Fig. 4b and c were tested using a Williams or Shirley-Williams test conducted at a one-tailed significance level of *P* < 0.025, based on results of the homogeneity of variance test (Bartlett’s test). The Aspin-Welch test or Student’s t-test between two groups, and Dunnett’s multiple-comparison test were performed at a significance level of *P* < 0.05 to analyse for statistical significance in Fig. 4d. All statistical analyses were performed using Statistical Analysis System version 9.3 (SAS Institute, NC, USA).

### Data availability

All datasets generated and/or analysed during the current study are available from the corresponding author on reasonable request.

## Electronic supplementary material

Supplementary information
