# Supplementary material for: Effects of 4(1H)-quinolinone derivative, a novel non-nucleotide allosteric purinergic P2Y2 agonist, on cardiomyocytes in neonatal rats
Source: Sci Rep. 2017 Jul 20;7:6050. doi: 10.1038/s41598-017-06481-9 (PMC5519634; doi:10.1038/s41598-017-06481-9)
Supplement: Supplementary file 1 — Supplementary information [file 41598_2017_6481_MOESM1_ESM.pdf]

Effects of 4(1*H*)-quinolinone derivative, a novel non-nucleotide allosteric purinergic $P2Y_2$ agonist, on cardiomyocytes in neonatal rats

Kensuke Sakuma, Hideyuki Nakagawa, Tatsuo Oikawa, Masakuni Noda, and Shota Ikeda

Supplementary Information

Materials and Methods

cAMP accumulation assay

To measure intracellular cAMP levels in parental 1321N1 cells in Supplementary Fig. S2, a cAMP-Screen system (Thermo Fisher Scientific Japan) was used according to the attached manual. Cultured cells were stimulated with ligands diluted in HBSS buffer containing 0.2 mM 3-isobutyl-1-methylxanthine (IBMX; Sigma-Aldrich, Japan) and 0.1% bovine serum albumin (Wako) at 37 ºC for 30 min, and then dissolved in lysis buffer. Lysates were incubated with cAMP-alkaline phosphatase conjugate and anti-cAMP antibodies on a secondary antibody–coated plate, followed by incubation with a CSPD/Sapphire-II RTU substrate/enhancer solution. Signals were read using an EnVision plate reader (Thermo Fisher Scientific).

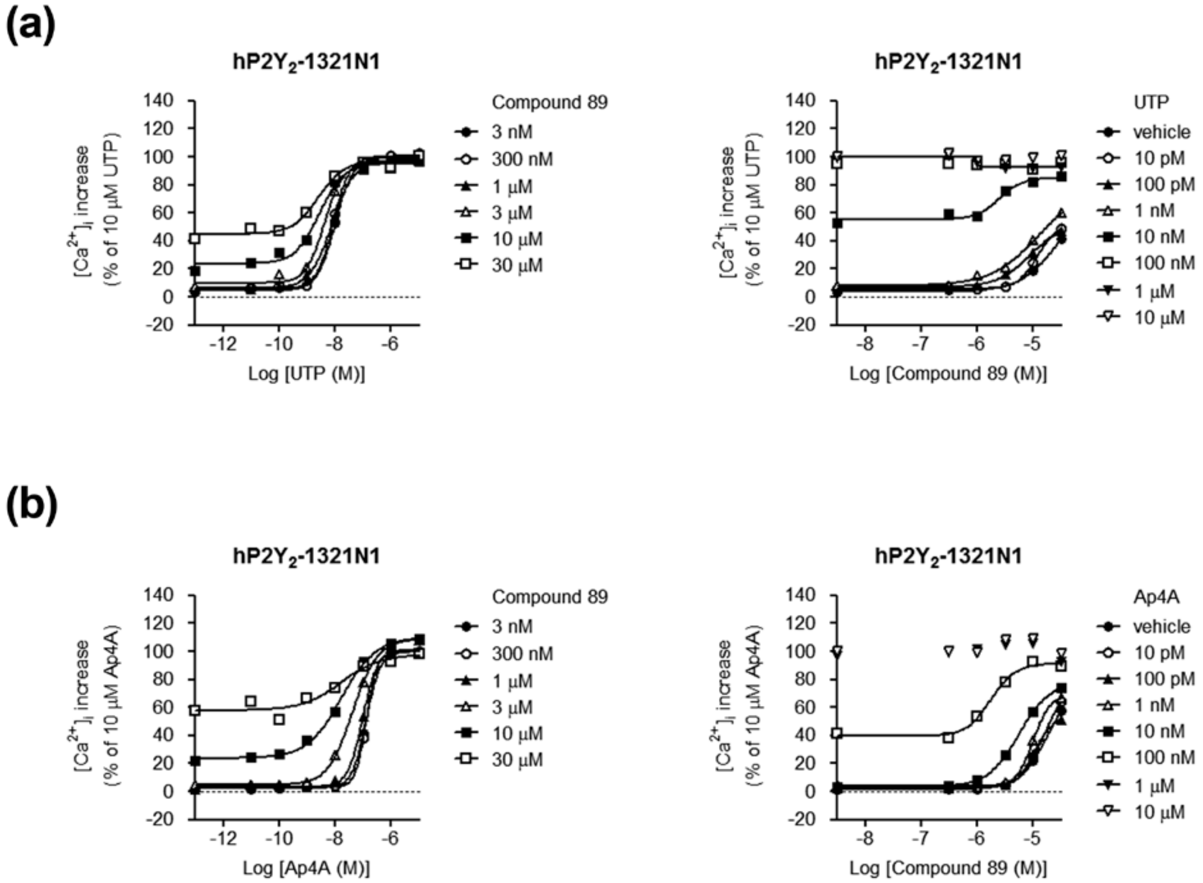

Supplementary Figure S1. Compound 89 behaves as an allosteric agonist towards the physiological agonists UTP and Ap4A in $P2Y_2$-overexpressing 1321N1 cells.

(a) Allosteric modulatory effect of Compound 89 on the UTP concentration-response curve (*left*), and allosteric modulatory effect of UTP on the Compound 89 concentration-response curve (*right*) and **(b)** Allosteric modulatory effect of Compound 89 on the diadenosine tetraphosphate (Ap4A) concentration-response curve (*left*), and allosteric modulatory effect of Ap4A on the Compound 89 concentration-response curve (*right*) in $hP2Y_2$ stably-expressing 1321N1 cells. All data points represent the

mean of experiments performed in duplicate, and comparable results were obtained from another independent experiment.

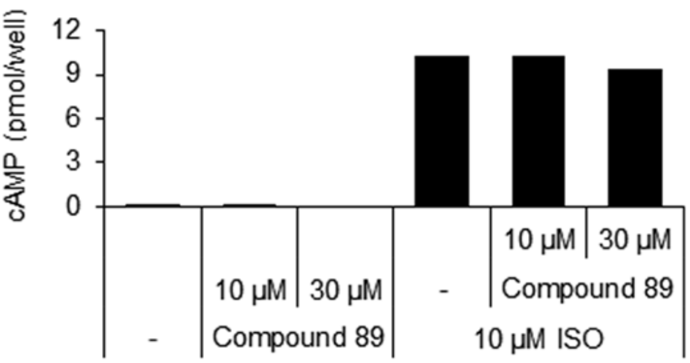

Supplementary Figure S2. Compound 89 does not affect β-adrenergic signalling in parental 1321N1 cells.

The effects of Compound 89, isoproterenol (ISO), and their combination on cAMP signalling in parental 1321N1 cells. All data points represent the mean of experiments performed in duplicate, and comparable results were obtained from another independent experiment.
